# Repellency, Toxicity, and Chemical Composition of Plant Essential Oils from *Myrtaceae* against Asian Citrus Psyllid, *Diaphorina citri* Kuwayama (Hemiptera Liviidae)

**DOI:** 10.3390/molecules29143390

**Published:** 2024-07-18

**Authors:** Yi-Jie Li, Tian-Ao Liu, Hang Zhao, Yang Han, Bing-Hai Lou, Cui-Yun Lei, Ya-Qin Song, Hong-Bo Jiang

**Affiliations:** 1 Guangxi Key Laboratory of Germplasm Innovation and Utilization of Specialty Commercial Crops in North Guangxi, Guangxi Academy of Specialty Crops, Guilin 541004, China; best116@foxmail.com (Y.-J.L.); meliodasssss@163.com (Y.H.); leicuiyun2016@163.com (C.-Y.L.); wrongpiano@163.com (Y.-Q.S.); 2 Guangxi Citrus Breeding and Cultivation Technology Innovation Center, Guangxi Academy of Specialty Crops, Guilin 541004, China; 3 Key Laboratory of Entomology and Pest Control Engineering, College of Plant Protection, Southwest University, Chongqing 400715, China; mayrain_lta@163.com (T.-A.L.); zhaohang990117@163.com (H.Z.); jhb8342@swu.edu.cn (H.-B.J.); 4 International Joint Laboratory of China-Belgium on Sustainable Crop Pest Control, Academy of Agricultural Sciences, Southwest University, Chongqing 400715, China

**Keywords:** botanical insecticides, GC-MS, insecticidal activity, Eucalyptus, chemical constituents

## Abstract

*Diaphorina citri* Kuwayama (*D*. *citri*) is one of the major pests in the citrus industry, which spreads Citrus Huanglongbing disease. It has developed resistance to chemical insecticides. Therefore, searching for greener solutions for pest management is critically important. The main aim of this study was to evaluate the repellent and insecticidal efficacy of essential oils (EOs) from four species of *Myrtaceae* plants: *Psidium guajava* (PG), *Eucalyptus robusta* (ER), *Eucalyptus tereticornis* (ET), and *Baeckea frutescens* (BF) against *D. citri* and to analyze their chemical compositions. GC-MS analysis was performed, and the results indicated that the EOs of PG, ER, ET, and BF were rich in terpenoids, ketones, esters, and alcohol compounds. The repellent rate of all four EOs showed that it decreased with exposure time but increased with the concentration of EOs from 80.50% to 100.00% after treating *D. citri* for 6 h with four EOs at 100% concentration and decreased to 67.71% to 85.49% after 24 h of exposure. Among the compounds from the EOs tested, eucalyptol had the strongest repellent activity, with a 24 h repellency rate of 100%. The contact toxicity bioassay results showed that all EOs have insecticidal toxicity to *D. citri*; the LC50 for nymphs was 36.47–93.15 mL/L, and for adults, it was 60.72–111.00 mL/L. These results show that when PG is used as the reference material, the ER, ET, and BF EOs have strong biological activity against *D. citri*, which provides a scientific basis for the further development of plant-derived agrochemicals.

## 1. Introduction

The Asian citrus psyllid, *Diaphorina citri* Kuwayama (Hemiptera: Psyllidae), is a globally important citrus pest. It feeds on leaf sap, causing leaf wilting and excreting on the leaves, leading to sooty mold pollution. Above all, it is a natural vector of Citrus Huanglongbing (HLB) [1,2]. HLB is a bacterial disease caused by the bacterium *Candidatus* Liberibacter asiaticus (CLas), which occurs in the phloem tissue of citrus plants [3,4]. Plants infected with CLas will gradually die within 2–4 years. Therefore, HLB has caused serious damage to the global citrus industry. Due to the current inability to cure HLB, controlling the citrus psyllid has become the primary measure for managing this disease [5]. 

At present, the use of chemical pesticides is the main measure for controlling *D. citri* in the field [6]. However, the extensive use of agrochemicals has led to serious environmental problems and the development of insecticide resistance [7,8]. Three *D. citri* field populations in Florida, USA, had developed high levels of resistance to the neonicotinoid agent thiamethoxam [9]. The resistance multiples of *D. citri* adults and 4th instar nymphs in three field populations in Mexico to malathion were 345–432 times and to chlorpyrifos were 1424–2435 times, indicating extremely high levels of resistance [10]. Such a high resistance factor makes field control of citrus psyllids even more difficult. Therefore, it is of utmost urgency to develop environmentally friendly and non-agrochemical measures to control *D. citri*.

Plant-based natural products are a new research focus in agricultural pest control [11,12,13], including *D. citri*. Plant-derived bioactive compounds have advantages such as renewability, affordability, biodegradability, strong specificity, environmental friendliness, and no resistance to pests. They can be used as effective alternatives to chemical pesticides against pests of significant medical and veterinary importance, as well as in agriculture [14,15,16,17]. Moreover, some plants can emit highly volatile and irritating odor substances, which have a significant repellent effect on pests. Previous studies have shown that the odor substances of PG could have a strong repellent effect on *D. citri* [18,19,20]. Additionally, some natural products of plants also have a certain killing effect on pests, and they do not cause resistance or pollution to the environment, offering a new environmentally friendly way of pest control [21]. 

*Psidium guajava* (PG) is considered an important tropical fruit, widely distributed in tropical and subtropical regions. PG fruit is very rich in nutritional elements and has been introduced into citrus production areas in southern China [22]. *Eucalyptus robusta* (ER) and *Eucalyptus tereticornis* (ET) are dense shade trees, both native to Australia and widely distributed in citrus-producing areas in southern China [23,24]. They are important timber plants, and their leaves can be used for medicine and have fumigation properties. *Baeckea frutescens* (BF) is a small shrub, mainly distributed in subtropical regions and also found in southern China. Its leaves have a volatile odor and can be used as medicine [25]. The leaves of these four plants all contain volatile substances, thus having the potential to be used as plant-based pesticides for pest control.

The prevention and control of citrus psyllid in orchards requires a combination of multiple measures to achieve optimal results [26]. Therefore, using a plant-based natural product as a repellent to control *D. citri* is a novel strategy. In this study, we screened four plants PG, ER, ET, and BF, extracted their essential oils (EOs) through distillation, and tested their repellent and insecticidal efficiency against *D. citri* through bioassay experiments. Then, we identified the compound components of these four EOs, selected the major small molecule compounds with the highest content in each EO, and conducted molecular docking experiments using DcitOBP7 from *D. citri* as the macromolecular target protein. Finally, behavioral experiments were conducted again using small molecule compounds with lower binding energies to support the results of molecular docking. We hope to develop new plant-based natural product repellents through this study, providing new strategies for non-pesticide control of *D. citri*. 

## 2. Result

### 2.1. Repellent Bioassay of EOs

**Figure 1:**
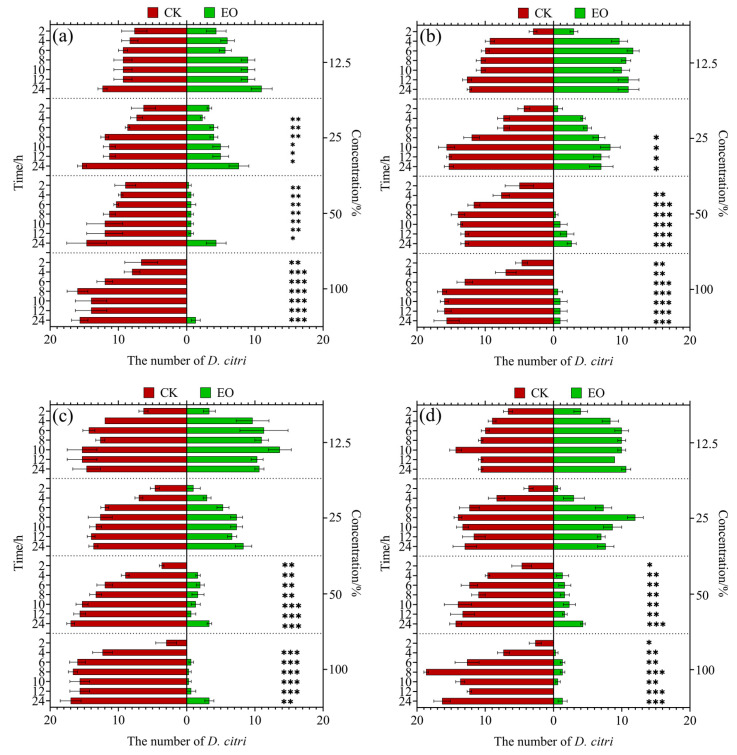
Comparison of the quantity of *Diaphorina citri* Kuwayama on tender shoots of *Murraya paniculata* with and without essential oil (EO) application. The red bar represents CK and the green bar represents EO. (**a**) PG. (**b**) ER. (**c**) ET. (**d**) BF. * *p* < 0.05, ** *p* < 0.01 and *** *p* < 0.001.

**Figure 2:**
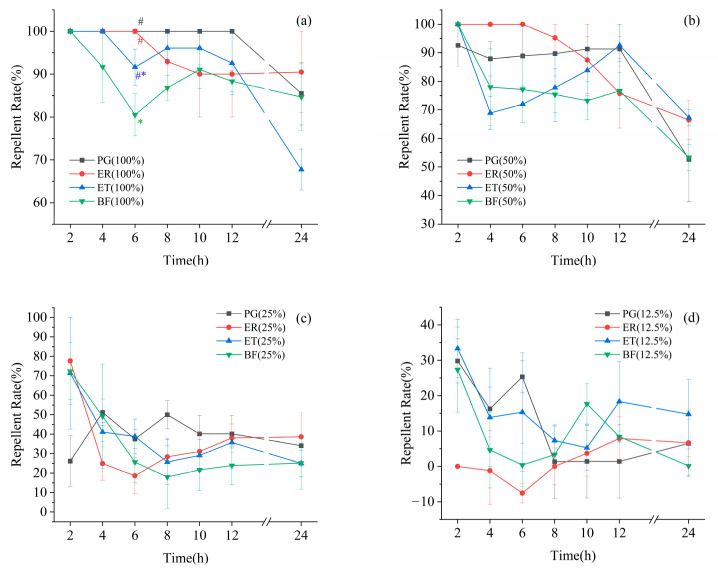
Analysis of significant differences in the repellent rates of *Diaphorina citri*. (**a**–**d**) represent different concentrations as follows: 100%, 50%, 75%, and 12.5%. Note: # and * represent a statistically significant difference using one-way analysis of variance (HSD, *p* < 0.05).

A comparison of the distribution quantity of *D. citri* on EO and a control check (CK) of all EOs over time and concentration are shown in Figure 1, while the significant differences in their repellent rates are shown in Figure 2. When the concentration was 100% or 50%, there was a significant difference in the number of CKs and EOs selected by *D. citri* among all four EO treatments. When the concentration was 25%, there was no significant difference in the number of CKs and EOs selected by *D. citri* in the ER and ET EO treatments; ER only showed significant differences at 8, 10, 12, and 24 h after treatment, and PG only showed no significant difference at 2 h after treatment. When the concentration was 12.5%, there was no significant difference in the number of CKs and EOs selected by *D. citri* among all four EO treatments. Only at a concentration of 100% and after 4 h of treatment, there was a significant difference in the repellent rates of PG and BF towards *D. citri*, while there was no significant difference in the repellent rates of the other four EOs at the same concentration and time period.

### 2.2. Toxicity Bioassay

The toxicity data of citrus psyllids treated with all EOs for 24 h are provided in Table 1. The toxicity bioassay results showed that the LC_50_ of BF EOs on nymphs and adults were 36.47 mL/L and 60.72 mL/L, respectively, with the best effect. For PG, the LC_50_ values were 93.15 mL/L for nymphs and 111.00 mL/L for adults. ER exhibited LC_50_ values of 53.85 mL/L for nymphs and 90.44 mL/L for adults, while ET showed LC_50_ values of 56.50 mL/L for nymphs and 77.19 mL/L for adults. The mortality rate of all EOs on nymphs was generally higher than that of adults.

### 2.3. Chemical Analysis of the EOs

The chemical compositions of the EOs from the four plants are provided in Table 2. A total of 121 compounds were identified from the four EOs. Terpenoids were the main components in all four plant EOs, accounting for 65.31%, 44.00%, 46.91%, and 46.15%, respectively. Additionally, the compound with the highest content in PG was β-cubebene (9.42%), in ER was α-phellandrene (12.20%), in ET was α-pinene (15.59%), and in BF was o-cymine (13.62%). β-caryophyllene, which was considered the main repellent component of PG, was present in PG (6.15%), ER (1.04%), ET (0.72%), and BF (2.89%), with the highest content in PG.

### 2.4. Repellent Bioassay of Compounds

Compounds with higher concentrations of various EOs were used to test their repellent activity. Dimethyl disulfide is often used as a positive control of olfactory experiment content, so we chose it as the positive control, and the results are shown in Table 3. Within 6 h of treatment, β-caryophyllene maintained a 100% repellent rate, while it decreased to 83.23–94.07% from 8 to 24 h. The compound α-pinene showed 100% effectiveness within 4 h and decreased to 76.92–93.33% from the 6th to the 24th hour. Eucalyptol maintained a high repellent effect for 24 h, which was 100% except for the 6th hour (94.86%) and the 8th hour (92.22%). The compound o-cymine exhibited 100% efficacy within 4 h and decreased to 54.77–91.91% within 6 to 24 h. The repellent rates of limonene and (R)-(+)-limonene within 24 h were −17.32–12.87% and −11.27–11.44%, respectively, with more attractive effects.

### 2.5. Molecular Docking

**Figure 3:**
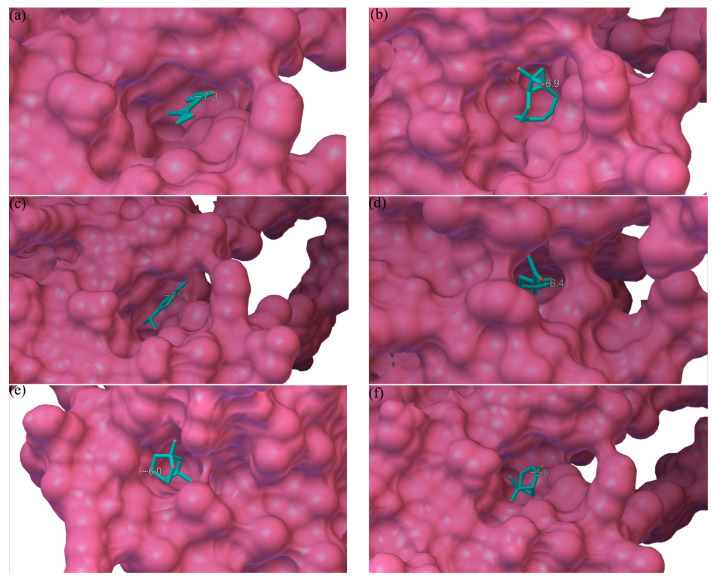
Molecular docking results of six ligands with DcitOBP7. (**a**) α-Pinene. (**b**) β-Caryophyllene. (**c**) α-Terpinene. (**d**) Limonene. (**e**) Eucalyptol. (**f**) β-Pinene.

The main role of DcitOBP7 in *D. citri* is as a high representative sensory protein; so in this study, we adopt DcitOBP7 receptor proteins for molecular docking. In order to explore the mechanism of action of the active compounds, six compounds with significant repellent activity were further tested for their binding abilities with DcitOBP7. And the results of molecular docking are shown in Figure 3, which illustrates the compounds’ strong binding affinity to the protein pocket with a noteworthy docking score ranging between −5.9 and −7.3 kcal/mol. For all the docking analyses, a lower score indicated a better binding affinity. The molecular docking analysis revealed that α-pinene had the best binding affinity at −7.3 kcal/mol with DcitOBP7. The compounds α-pinene, β-caryophyllene, α-terpinene, and β-pinenewere docked at the same position as DcitOBP7, while limonene and eucalyptol were docked in different positions.

## 3. Discussion

The olfactory receptor of insects is an important system that regulates their behaviors, such as foraging, searching for mates, mating, laying eggs, and avoiding natural enemies [27,28]. Odorant binding proteins (OBPs) are one of the olfactory proteins in insects, and among various olfactory proteins in insects, they play a major role in the perception of odor factors [29,30]. OBPs are water-soluble macromolecular proteins. When insects sense and recognize odors in the environment, OBPs are responsible for binding and transporting these odorants [28]. Because of their role in insect signal transduction, OBPs are considered significant research targets for pest control [31]. OBPs had been identified from various Hemipteran insects, such as *Acyrthosiphon pisum* (15 OBPs) [32], *Sogatella furcifera* (12 OBPs) [33], *Bemisia tabaci* (8 OBPs) [34], and *D. citri* (9 OBPs) [35].

In order to simulate the living habits of citrus psyllids and the actual situation in orchards, this study used arranged tender shoots in cages to test the repellent activity of EOs. So far, several studies have been conducted on the repellent efficiency of PG against citrus psyllids. Gottwald et al. [36] indicated that intercropping PG with citrus could reduce the infestation rate of citrus psyllids by 50–100% compared to planting citrus alone. Zaka et al. [20] indicated that when PG leaves were around citrus leaves, the feeding quantity of citrus psyllids decreased by 36.62% to 52.70%. Therefore, guava volatile oil could be regarded as a positive control with good effects. Indoor repellent tests showed that the repellent rate of PG against citrus psyllids within 24 h was 85.49–100%, which was sufficient to confirm the significant repellent effect of PG on citrus psyllids. Additionally, EOs from ER, ET, and BF exhibited similar repellent effects on citrus psyllids within 24 h, as guava oil. Significance test results indicated that, except for the 100% concentration at 6 h and 12.5%, there was no significant difference in the repellent efficiency under the same concentration and time conditions. Among all EOs, EO from ER could maintain a high repellent rate even at low concentrations and exhibit long-term effects. ER, ET, and BF were all distributed in southern China, overlapping with the main citrus-producing areas. This suggested that ER, ET, and BF all had the potential to be used as plant-based pesticides for *D. citri* repellent.

Through GC-MS analysis, a total of 121 compounds were identified. Subsequently, highly abundant and commercially available compounds were used for the test of repellent activity, individually. Table 3 showed that some compounds could significantly repel *D. citri*, while others had no significant repellent effect on psyllids, and even had a certain attractive effect. β-caryophyllene is one of the main chemical constituents of PG EO, and its effectiveness in repelling *D. citri* has been confirmed [37]. In this study, β-caryophyllene also exhibited good repellent activity, with a repellent rate of 85.00 ± 4.28% after 24 h. The composition of the other three plant EOs was significantly different from that of PG, but they all had a repellent effect, similar to that of PG EO. This might be closely related to α-pinene and eucalyptol. It was worth noting that eucalyptol showed significant repellent activity during testing, with a 24 h repellent rate of 100%. Eucalyptol was not detected in PG EO, while the relative content in EOs of ER, ET, and BF was 5.91%, 6.87%, and 4.31%, respectively. This indicated that eucalyptol might be the major active constituent of the EOs extracted from these three plants.

In toxicity bioassays, the mortality rate of nymphs was higher than that of adults. This was because, after soaking the leaves, the EOs formed an oil film on the surface of the plant leaves, hindering the feeding of insects. At the same time, EOs could clog the pores of insects and cause them to suffocate. Their preventive and control effects were similar to those of mineral oil pesticides [38]. Compared to adults, nymphs had softer mouthparts, which were more difficult to penetrate plant leaves covered with oil film for feeding. In addition, the nymphs’ tolerance to food shortage and respiratory restriction was significantly lower than that of adults. Therefore, using the EOs from the four natural plants in this study could repel adult insects with flight ability and eliminate nymphs with weaker activity levels, providing a theoretical basis for the development of new *D. citri* repellents and insecticides [39].

The docking results of the DcitOBP7 molecule showed that both the central and edge regions of DcitOBP7 had hydrophobic pocket-like cavities, which provided a possibility for the binding of various ligands to DcitOBP7. The results of molecular docking experiments revealed that the top three ligands with the lowest binding energies, α-pinene, β-caryophyllene, and limonene, could all be embedded into the central hydrophobic pocket-like cavity of DcitOBP7 [40]. In addition, eucalyptol, which had a stronger tendency to repel *D. citri*, could be placed in the hydrophobic pockets at the center and edge of DcitOBP7, respectively, and there were few other ligands embedded at the docking sites located at the edge, which created conditions for eucalyptol to achieve diversified docking on DcitOBP7.

In comparison with experimental results from molecular docking and compound repellent rate determinations, although the lowest binding energy of eucalyptol and DcitOBP7 docking was higher than that of the other four compounds, it had a separate docking site at the edge of DcitOBP7. Additionally, in behavioral experiments, eucalyptol showed stronger persistence compared to other compounds and still had a significant effect on *D. citri* after 24 h. Moreover, the compound had lower corrosiveness to plant leaves, and after 24 h of use, the leaves of *M. paniculata* could still maintain a fresh green state. Eucalyptol itself had insecticidal activity and was used to kill insects. Research has shown that eucalyptol had an impact on *M. Domestica* and *C. Megacephala* has toxicity [41,42,43]. Therefore, eucalyptol had high potential as both a plant-based pesticide and a repellent. However, due to the toxicity of eucalyptol to mammals, attention should be paid to its safety issues in practical applications [44,45]. Compared with eucalyptol, α- pinene had the lowest binding energy, and experimental results also indicated that within 12 h, α- pinene has a strong repellent effect. However, its repellent rate would significantly decrease after 24 h. Consequently, this compound was not suitable for use as a repellent alone.

In the previous study reported by María et al. [46], limonene showed a significant attractive effect on *D. citri*. In this study, we found that limonene exhibited a maximum attractive effect of 17.32% on *D. citri*, which was consistent with María et al.’s report. D-limonen also had an attraction effect of 11.27% on citrus psyllids. Therefore, limonene has the potential to be developed as a new *D. citri* attractive agent. In subsequent experiments, we will continue to conduct research on the effectiveness of attractive agents.

This study once again confirmed the repellent effect of PG OE on *D. citri.* PG is rich in various vitamins, such as vitamin C and mineral elements, which can effectively promote the synthesis of nitric oxide in the human body, and have the effects of dilating blood vessels and lowering blood pressure [47,48]. The high content of β-caryophyllene in PG leaves can also be used as a pest repellent and attractant in agricultural pest control.

As a widely planted and vigorous plant, ER has advantages such as rapid growth, abundant yield, and outstanding carbon sequestration capacity. In southern China, especially in citrus-producing areas, ER has become an important forestry resource [49]. Therefore, using ER as raw material to develop a natural plant repellent targeting *D. citri* will greatly enhance the economic value of ER, alleviate the pressure of *D. citri* prevention and control, and slow down the growth of *D. citri* resistance. In addition to providing plant EOs, planting ER around citrus orchards can establish repellent isolation zones, thereby blocking the flight of *D. citri*. This will also provide a basis for green prevention and control of *D. citri* in the field.

## 4. Materials and Methods

### 4.1. Plant and Insect Materials

#### 4.1.1. Plant Materials

Fresh twigs and leaves from four kinds of plants, PG, ER, ET, and BF, were brought from a local market. The sample was stored in a moist environment at 4 °C after collection and extracted and processed within 6 h. Plant samples were kept in the laboratory of Guangxi Academy of Speciality Crops.

#### 4.1.2. Insect Materials

*D. citri* were raised in the Insect Laboratory of Guangxi Academy of Specialty Crops (110°18′51″ E, 25°5′18″ N), and 300 healthy *M. paniculata* plants were planted in a netted area within the greenhouse (25 ± 2 °C, 70 ± 10% relative humidity, photoperiod of 16 h light: 8 h dark) as a food source for them. We chose the first generation of all *D. citri*, without any toxic test strain experiment.

### 4.2. Extraction of the EOs

Plant EOs were extracted using the steam distillation method. Plant samples were ground into powder and subjected to steam distillation using a Clevenger-type apparatus for 2 h. The collected oil samples were dried over anhydrous Na_2_SO_4_ and stored at 4 °C for further analysis.

### 4.3. Repellent Bioassay of EOs

A solution of 25% acetone-aqueous solution was used to dilute EOs. The EOs were set with concentration gradients of 100%, 50%, 25%, and 12.5%. Thirty *D. citri* adults were placed in plastic pipes (h = 10 cm, d = 1.5 cm) under a hunger treatment for 6 h. Two healthy young shoots of *M. exotica* (About 5 to 7 cm) were cut and inserted into two plastic tubes filled with water; one of the shoots had evenly applied EOs and the other had applied water. The two tubes with shoots were placed at opposite corners in a net cage (60 cm × 60 cm × 60 cm). The *D. citri* that had completed hunger treatment were released in the center of the cage where there was an equal distance to the two tubes with shoots; each of the EOs and all its dilution gradients were tested. There were three independent replicates for each treatment. All the treated *D. citri* were maintained in an incubator (25 ± 2 °C, 70 ± 10% RH with a 14:10 h L:D photoperiod), and the number of *D. citri* on different treatment shoots was checked after being treated for 2, 4, 6, 8, 10, 12, and 24 h. The repellent efficiency was calculated using the following formula:

$$\mathrm{Repellent}\;\mathrm{Rate}\%=\frac{Nc-Ne}{Nc+Ne}\;\text{×}\;100\%$$

where *Nc* is the number of *Diaphorina citri* that chose control shoots; and *Ne* is the number of *D. citri* that chose essential oils.

### 4.4. Toxicity Bioassay

The toxicity bioassay of *D. citri* was performed using a leaf dip bioassay method. The EOs were diluted with a 25% acetone-water solution in different concentrations. For each EO, *M. paniculata* leaves were immersed for 10 s in essential oils and their dilute solutions and in 25% acetone-water solution (controls). The leaves were air-dried for 30 min before being placed individually in a plastic cup (h = 20 cm, d = 5 cm). After the leaves had dried, thirty *D. citri* adults and nymphs were placed on them. There were three independent replicates in each treatment. All the treated *D. citri* were maintained in the incubator (25 ± 2 °C, 70 ± 10% RH with a 14:10 h L:D photoperiod), and the number of deaths in *D. citri* on different treatment leaves was checked after 24 h of treatment.

### 4.5. Composition Analysis of the EOs by GC-MS

The composition of EOs was analyzed by gas chromatography coupled to mass spectrometry (GC-MS) Agilent Model 8890 GC and a 7000D mass spectrometer (Agilent, Santa Clara, USA), equipped with a 30 m × 0.25 mm × 0.25 μm DB-5MS capillary column. Helium was used as the carrier gas with a linear velocity of 1.2 mL/min. The injector temperature was maintained at 250 °C. The oven temperature was programmed from 40 °C 3.5 min, increasing at 10 °C/min to 100 °C, then at 7 °C/min to 180 °C, and finally at 25 °C/min to 280 °C and held for 5 min. Mass spectra were recorded in electron impact (EI) ionization mode at 70 eV. The quadrupole mass detector, ion source, and transfer line temperatures were set, respectively, at 150, 230, and 280 °C. The MS was operated in selected ion monitoring (SIM) mode for the identification of analyses. The chemical constituents were identified by comparing their mass spectra alongside the linear retention indices using those from the NIST20 database and the consulted/existing literature. Relative abundance percentages of individual compounds were quantified as the average peak area percentages, without using correction factors.

### 4.6. Repellent Bioassay of Compounds

Based on the results of Section 4.5, the main compounds in EOs were selected and subjected to repellent bioassay. The bioassay method was the same as in Section 4.3, but it did not involve a designed concentration gradient. The compounds β-caryophyllene, terpinene, β-pinene, linalool, eucalyptol, α-pinene, phellandrene, ocimene, D-limonene, γ-terpinene, o-cymene, cineole, 1,4-diethylbenzene, limonene, 3-carene, 1-phenylhexan-3-one, and myrtol were purchased from Macklin Chemical Reagent Co., Ltd. (Shanghai, China). 

### 4.7. Molecular Modeling and Docking

The tertiary structure of DcitOBP7 was modeled using the AlphaFold2 v2.3.0 software. The 3D structure of ligands was downloaded in the PubChem database (http://pubchem.ncbi.nlm.nih.gov/ accessed on 15 July 2023). Molecular docking was performed using AutoDock Vina 1.2.0 and visual analysis of molecular docking results was conducted using AutoDockTools-1.5.7.

### 4.8. Statistical Analysis

The Nonparametric tests for related samples were used to analyze differences in the number of *D. citri* on shoots processed differently. One-way ANOVA was used to analyze differences in repellent rates between different plant EOs and different compounds. Data are shown as mean values ± standard error of the mean (SEM). The *p* values < 0.05 were considered statistically significant. All statistical analysis was performed using SPSS version 22.0 software.

## 5. Conclusions

In conclusion, this study reports the repellent activity of EOs from four plants PG, ER, ET, and BF. In the EOs repellent experiment, the feeding selectivity of citrus psyllids treated with starvation was used as a criterion to evaluate the repellent efficiency. Based on this evaluation standard, all four tested plant EOs have significant repellent efficiency against citrus psyllid. Through molecular docking and compound-repellent experiments, we have identified several compounds that are sensitive to citrus psyllids and have high repellent efficiency, just like β-caryophyllene, α-pinene, and eucalyptol, which can provide a basis for the prevention and control of citrus psyllids.

## Figures and Tables

**Table 1:** Toxicity of plant essential oils (EOs) against workers of *Diaphorina citri* at 24 h post-treatment.

Stage	EOs	*n*	Slope *±* SEM	LC_50_ (95% CI)	LC_90_ (95% CI)	χ^2^	df	Control Group Death Rate%
Nymph	PG	90	1.80 ± 0.20	93.15 (77.13–115.63)	480.04 (326.26–861.10)	1.90	3	5.60
	ER	90	1.40 ± 0.18	53.85 (42.39–73.36)	441.24 (249.63–826.42)	1.31	3	6.70
	ET	90	1.11 ± 0.18	56.50 (41.76–86.80)	484.80 (357.29–602.69)	0.84	3	2.20
	BF	90	1.63 ± 0.17	36.47 (30.27–44.79)	222.12 (151.00–391.32)	3.06	3	7.80
Adult	PG	90	1.53 ± 0.16	111.00 (91.73–137.59)	766.79 (506.96–1008.07)	1.83	3	0.00
	ER	90	1.11 ± 0.15	90.44 (70.31–119.15)	777.67 (457.71–1084.19)	0.79	3	0.00
	ET	90	1.36 ± 0.15	77.19 (62.36–95.72)	680.89 (438.03–926.14)	0.23	3	0.00
	BF	90	1.63 ± 0.16	60.72 (50.22–72.60)	370.69 (270.22–578.70)	0.12	3	0.00

Note: LC_50_ and LC_90_ values were represented in mL/L and CIs were confidence intervals.

**Table 2:** Chemical composition of the four plants’ essential oils (EOs).

No.	Compounds	NIST RI	Relative Abundance (%)			
			PG	ER	ET	BF
**1**	4-Hexen-3-one	855	-	0.51	-	-
**2**	Dimethyl sulfone	922	-	0.60	3.02	-
**3**	Benzene, (1-methylethyl)-	926.57	-	-	1.68	-
**4**	Cyclobutanespiro-2′-bicyclo [1.1.0]butane-4′-spirocyclobutane	930	-	1.10	5.85	0.68
**5**	α-Pinene	936.35	-	3.40	15.59	3.21
**6**	Cyclopentene, 1-butyl-	938	-	-	0.93	-
**7**	Bicyclo (3.3.1)non-2-ene	964	-	-	0.68	-
**8**	4-methyl-1-(1-methylethyl)-Bicyclo[3.1.0]hex-2-ene	966	-	-	1.21	-
**9**	Bicyclo[3.1.0]hexane, 4-methylene-1-(1-methylethyl)-	972	-	0.45	1.62	-
**10**	2,6-Octadiene, 2,6-dimethyl-	978	-	0.43	1.44	-
**11**	β-Pinene	979.71	-	1.81	6.25	0.70
**12**	1,7-Octadiene, 2-methyl-6-methylene-	984	-	0.38	1.31	-
**13**	Disulfur compounds, ethyl 1-methylethyl	985	-	-	0.58	-
**14**	Pyridine, 3-propyl-	986	-	-	0.73	-
**15**	Benzene, (1-methylpropyl)-	1001	-	0.37	-	-
**16**	α-Phellandrene	1006	-	12.20	0.77	0.55
**17**	Terpilene	1018.03	-	-	-	-
**18**	4-Hexen-1-ol, acetate	1020	-	0.67	0.67	0.63
**19**	o-Cymene	1022	-	10.70	4.13	13.62
**20**	4,6-Octadiyn-3-one, 2-methyl-	1023	0.69	-	-	-
**21**	2-Azabicyclo[3.2.1]octan-3-one	1025	-	1.86	2.05	1.65
**22**	p-Cymene	1025.98	-	3.77	1.55	5.34
**23**	Limonene	1026	3.66	3.08	2.32	0.76
**24**	2-Methyl-1,3-dithiacyclopentane	1026	-	1.15	-	1.51
**25**	1,7-Nonadiene, 4,8-dimethyl-	1026	-	0.85	0.46	-
**26**	Thiazole, 5-ethenyl-4-methyl-	1027	-	0.76	0.93	0.63
**27**	Pyridine, 2,3,4,5-tetrahydro-6-propyl-	1028	-	0.56	0.65	-
**28**	Indane	1029	-	1.69	0.63	2.19
**29**	Cyclohexanol, 3,5-dimethyl-	1030	-	3.26	2.83	2.94
**30**	β-Phellandrene	1031	2.68	1.77	4.36	2.82
**31**	D-Limonene	1031.27	3.15	4.13	3.19	2.35
**32**	Eucalyptol	1034.33	-	5.91	6.87	4.31
**33**	3-Octen-2-one, (E)-	1035	-	3.23	3.92	2.89
**34**	Ocimene	1037	0.53	5.72	1.21	-
**35**	2-Acetyl-5-methylfuran	1037.22	-	0.50	0.57	-
**36**	(S)-2,5-Dimethyl-3-vinylhex-4-en-2-ol	1039	0.96	4.32	4.32	2.93
**37**	3-Octen-2-one	1040	-	1.89	1.40	1.55
**38**	Benzeneacetaldehyde	1045.59	-	0.48	-	-
**39**	(E)-β-Ocimene	1049	-	0.96	-	-
**40**	γ-Terpinene	1060.24	-	0.57	-	3.02
**41**	Benzenemethanol, α-methyl-	1061.21	-	-	-	0.94
**42**	trans-4-thujanol	1070	-	-	-	1.82
**43**	Benzaldehyde, 3-methyl-	1070.12	-	0.48	-	2.37
**44**	(Z)-Pent-2-enyl butyrate	1091	-	-	-	1.77
**45**	Linalool	1100.58	-	-	-	0.98
**46**	6-Nonenal, (Z)-	1103.52	-	-	-	1.03
**47**	Pinocarveol	1138	-	-	0.91	-
**48**	Myrcenone	1145	-	-	0.52	-
**49**	p-Mentha-1 (7),2-dien-8-ol	1163	-	-	0.70	-
**50**	Pinocarvone	1164	-	-	1.41	-
**51**	Phenol, 4-ethyl-	1165.40	-	-	0.63	-
**52**	(E)-2,6-Dimethylocta-5,7-dien-2-ol	1169	-	-	-	1.66
**53**	Lavandulol	1170	-	-	-	1.36
**54**	Borneol	1170.41	-	-	0.68	-
**55**	Terpinen-4-ol	1181.45	-	0.53	-	2.38
**56**	2-Butenoic acid, hexyl ester	1191	-	-	-	0.73
**57**	(-)-Dihydrocarveol	1192	-	-	-	0.56
**58**	α-Terpineol	1195.55	-	-	-	0.59
**59**	Benzamide	1344	-	0.46	-	-
**60**	2,3,5,9-tetramethyltricyclo[6.3.0.01,5]undec-3-ene	1348	-	4.20	-	-
**61**	Terpinyl acetate	1350	-	2.89	-	-
**62**	(1α,3β,4β)-p-menthane-3,8-diol	1355	-	0.61	-	-
**63**	Neryl acetate	1365.22	-	3.09	-	-
**64**	Methyl 4-aminobenzoate	1372	1.30	-	-	-
**65**	6,8-Nonadien-2-one, 8-methyl-5-(1-methylethyl)-, (E)-	1373	0.59	-	-	-
**66**	(-)-α-Copaene	1376	3.54	-	-	-
**67**	Di-epi-α-cedrene- (I)	1382	5.52	-	-	-
**68**	(-)-β-Bourbonene	1384	1.12	-	-	-
**69**	(-)-Modhephene	1385	0.63	-	-	-
**70**	Damascenone	1386	0.71	-	-	-
**71**	Acetic acid, phenoxy-	1389	0.79	-	-	-
**72**	β-Cubebene	1390	9.42	-	-	-
**73**	Niacinamide	1419	0.80	-	-	-
**74**	Ethyl mandelate	1421	-	0.69	0.51	1.98
**75**	Benzoic acid, 4-methoxy-	1424.27	1.49	-	-	-
**76**	Benzenemethanol, 4-hydroxy-	1426	1.42	-	-	0.61
**77**	3-Hexanone, 1-phenyl-	1427	5.14	0.87	0.60	2.34
**78**	2-Propenoic acid, 3-phenyl-	1427.53	-	-	-	1.04
**79**	Quinoxaline, 2,3-dimethyl-	1428	0.50	-	-	-
**80**	(E,E)-2,4-Undecadienal	1430	2.16	0.40	-	1.08
**81**	(+)-Calarene	1432	5.08	0.36	-	1.43
**82**	β-Caryophyllene	1432.49	6.15	1.04	0.72	2.89
**83**	γ-Elemene	1433	2.27	-	-	0.92
**84**	Ethyl β-safranate	1434	3.22	0.47	-	1.31
**85**	trans-α-bergamotene	1435	3.27	0.43	-	1.22
**86**	2-Hydroxymethylbenzimidazole	1437	3.14	-	-	0.96
**87**	Ethanone, 1-(3-hydroxyphenyl)-	1439	0.76	-	-	-
**88**	Azulene, 1,2,3,3a,6,8a-hexahydro-1,4-dimethyl-7-(1-methylethyl)-, (1R,3aS,8aS)-	1440	3.09	-	-	-
**89**	Naphthalene, 1,2,4a,5,8,8a-hexahydro-4,7-dimethyl-1-(1-methylethyl)-, (1α,4aβ,8aα)- (.+/−.)-	1440	3.34	-	-	0.99
**90**	Aromandendrene	1440	0.85	-	-	-
**91**	(+)-α-Muurolene	1440	-	0.62	-	1.67
**92**	Benzyl angelate	1446	1.25	-	-	-
**93**	-6-Methyl-2-methylene-6- bicyclo[3.1.1]heptane	1446	1.02	-	-	-
**94**	(-)-Aristolene	1447	2.08	-	-	-
**95**	Benzene, 1-(1,5-dimethylhexyl)-4-methyl-	1449	1.17	-	-	-
**96**	(-)-α-Himachalene	1449	1.11	-	-	-
**97**	Acetophenone, 4’-hydroxy-	1455	0.76	-	-	2.21
**98**	(E)-β-Famesene	1457	-	-	-	1.02
**99**	5,9-Undecadien-2-ol, 6,10-dimethyl-	1459	-	-	-	0.74
**100**	1,1′-(1,4-phenylene)bis-ethanone	1461	0.67	-	-	-
**101**	Benzene, [1-[[1-(1-methylethyl)-3-butenyl]oxy]ethyl]-, [S-(R*,R*)]-	1463	-	-	-	0.91
**102**	2-Pinen-10-yl isobutyrate	1466	-	-	-	1.46
**103**	(1R,9R,E)-4,11,11-Trimethyl-8-methylenebicyclo[7.2.0]undec-4-ene	1466	-	-	-	0.90
**104**	Acoradiene	1471	-	-	-	0.64
**105**	(4R,4aS,6S)-4,4a-Dimethyl-6-(prop-1-en-2-yl)-1,2,3,4,4a,5,6,7-octahydronaphthalene	1476	0.57	-	-	-
**106**	Eudesma-2,4,11-triene	1479	0.54	-	-	-
**107**	(-)-Germacrene D	1481	1.52	-	-	-
**108**	3-(4-Hydroxyphenyl)propanal	1490	-	0.37	1.01	-
**109**	(1S,2E,6E,10R)-3,7,11,11-tetramethylbicyclo[8.1.0]undeca-2,6-diene	1495	-	0.81	2.07	-
**110**	Benzyl tiglate	1498	-	-	0.68	-
**111**	α-Muurolene	1499	0.66	-	0.63	-
**112**	Epizonarene	1501	-	-	0.57	-
**113**	α-Cuprenene	1509	-	-	0.61	-
**114**	(E)-α-Bisabolene	1512	0.65	0.47	1.27	-
**115**	(-)-γ-Cadinene	1513	-	-	0.74	-
**116**	cis-Calamenene	1523	3.01	-	-	-
**117**	(+)-δ-Cadinene	1524	0.64	-	-	-
**118**	Cadinadiene,cadina-1,4-diene	1532	0.92	-	-	-
**119**	(+)-α-Cadinene	1538	0.89	-	-	-
**120**	β-Vetivenene	1540	0.50	-	-	-
**121**	3,7 (11)-Eudesmadiene	1542	1.29	-	-	-
	Total		97.21	97.89	97.97	96.79
	Terpineoids		65.31	44.00	46.91	46.15
	Ketone		12.24	10.00	8.16	9.61
	Ester		6.12	8.00	8.16	9.61
	Alcohol		4.08	6.00	6.12	13.46
	Acid		4.08	5.00	-	1.92
	Hydrocarbons		-	6.00	12.24	1.92
	Heterocyclic compound		4.08	8.00	8.16	5.77
	Aromatics		-	6.00	6.12	5.77
	Amine		2.04	2.00	-	-
	Aldehyde		2.04	8.00	2.04	5.77

Note: “-” means not detected.

**Table 3:** Analysis of differences in repellent rates of different compounds on *Diaphorina citri* at the same time.

Time (h)		2	4	6	8	10	12	24
Compounds	CAS	Mean ± SEM%						
β-Caryophyllene	87-44-5	100 ± 0.00	100 ± 0.00	100 ± 0.00	94.07 ± 2.97 a	83.23 ± 5.2 abc	83.23 ± 5.2 ab	85.00 ± 4.28 ab
α-Terpinene	99-86-5	100 ± 0.00	100 ± 0.00	85.05 ± 7.87 abc	67.72 ± 2.69 abcd	70.61 ± 10.32 abcd	68.15 ± 10.76 bcd	80.37 ± 1.61 abc
β-Pinene	127-91-3	80.61 ± 11.56 abc	50.27 ± 8.93 bc	42.06 ± 4.83 def	58.36 ± 12.59 cde	60.69 ± 5.52 bcd	73.45 ± 4.92 abc	72.01 ± 5.01 abc
Linalool	78-70-6	55.19 ± 2.89 bc	69.11 ± 1.38 ab	62.29 ± 4.85 bcd	54.94 ± 1.6 def	52.31 ± 1.31 cd	52.98 ± 0.79 cd	53.7 ± 0.85 c
Eucalyptol	470-82-6	100 ± 0.00	100 ± 0.00	94.86 ± 2.57 ab	92.22 ± 4.01 ab	100 ± 0.00	100 ± 0.00	100 ± 0.00
α-Pinene	80-56-8	100 ± 0.00	100 ± 0.00	80.43 ± 5.66 abc	86.98 ± 3.49 abc	93.33 ± 6.67 a	87.13 ± 3.57 ab	76.92 ± 4.47 abc
Phellandrene	99-83-2	100 ± 0.00	77.78 ± 11.11 ab	83.07 ± 5.82 abc	93.65 ± 6.35 a	84.13 ± 11.45 ab	72.26 ± 5.56 abcd	52.84 ± 2.47 c
Ocimene	13877-91-3	4.58 ± 17.56 de	−14.31 ± 9.51 e	−9.39 ± 4.13 h	−4.32 ± 2.27 hi	−4.32 ± 2.27 e	−4.53 ± 2.43 f	7.34 ± 6.82 d
D-Limonen	5989-27-5	−9.16 ± 2.38 e	−11.27 ± 4.36 e	−8.43 ± 1.56 h	−8.43 ± 3.24 i	−8.97 ± 2.19 e	11.44 ± 3.23 f	2.7 ± 1.96 d
γ-Terpinene	99-85-4	48.89 ± 14.57 bcd	26.83 ± 8.04 cd	28.92 ± 1.31 defg	25.41 ± 4.81 fgh	43.39 ± 10.62 d	53.33 ± 4.63 cd	20.08 ± 6.76 d
o-Cymene	527-84-4	100 ± 0.00	100 ± 0.00	91.91 ± 4.05 ab	71.42 ± 5.72 abcd	70.98 ± 5.49 abcd	63.14 ± 1.57 bcd	54.77 ± 5.29 c
Cineole	406-67-7	100 ± 0.00	65.02 ± 3.37 abc	45.95 ± 2.57 def	63.24 ± 7.25 bcde	48.03 ± 4.16 d	79.35 ± 10.62 abc	65.72 ± 9.22 bc
1,4-Diethylbenzene	105-05-5	−1.06 ± 7.35 e	4.15 ± 9.54 de	−9.09 ± 4.29 h	−1.45 ± 1.45 hi	1.15 ± 1.15 e	1.76 ± 7.54 f	9.70 ± 5.78 d
Limonene	138-86-3	−10.82 ± 10.64 e	−17.32 ± 8.52 e	−9.09 ± 4.29 h	−9.09 ± 4.29 i	−9.09 ± 4.29 e	12.87 ± 3.25 f	3.20 ± 1.62 d
3-Carene	13466-78-9	8.91 ± 13.76 cde	1.14 ± 5.46 de	1.42 ± 3.34 gh	−10.07 ± 4.36 i	−9.97 ± 4.43 e	11.42 ± 3.1 f	3.70 ± 6.42 d
1-Phenylhexan-3-one	29898-25-7	8.38 ± 16.9 de	1.63 ± 8.13 de	17.21 ± 12.83 fgh	5.41 ± 1.84 hi	5.41 ± 1.84 e	16.76 ± 3.32 ef	21.56 ± 1.52 d
Myrtol	8002-55-9	65.02 ± 3.37 ab	39.09 ± 5.09 bcd	23.74 ± 4.27 efgh	12.27 ± 2.58 ghi	12.27 ± 2.58 de	12.27 ± 2.58 f	21.43 ± 4.59 d
N		3	3	3	3	3	3	3
df		16	16	16	16	16	16	16
X^2^		24.532	44.98	45.54	46.16	44.81	45.07	45.337
*p*-Value		<0.001	<0.001	<0.001	<0.001	<0.001	<0.001	<0.001

Note: Different letters represent a statistically significant difference using Nonparametric tests for related samples (HSD, *p* < 0.05).

## Data Availability

The original contributions presented in the study are included in the article, further inquiries can be directed to the corresponding author.
